# Monomorphic epitheliotropic intestinal T-cell lymphoma presenting as acute small intestinal perforation: a case report and exploratory BCL-2–targeted therapy

**DOI:** 10.3389/fmed.2026.1818999

**Published:** 2026-06-05

**Authors:** Meijuan He, Yao Zhou, Jia Yao, Qiu Zhang, Wenjing Xu, Xiaoping Ye, Yihan Cai, Zefa Liu

**Affiliations:** 1 Department of Hematology, Xinghua People's Hospital Affiliated to Yangzhou University, Taizhou, Jiangsu, China; 2 Department of Oncology, Xinghua People's Hospital Affiliated to Yangzhou University, Taizhou, Jiangsu, China; 3 Shanghai DIAN Medical Laboratory Co., Ltd., Shanghai, China; 4 Nanjing Dian Diagnostics Group Co., Ltd., Nanjing, China

**Keywords:** case report, CHOP regimen, gastrointestinal perforation, monomorphic epitheliotropic intestinal T-cell lymphoma, targeted therapy, Venetoclax

## Abstract

**Objective:**

To report a rare case of monomorphic epitheliotropic intestinal T-cell lymphoma (MEITL) presenting with acute gastrointestinal perforation and to describe the use of an exploratory regimen combining a BCL-2 inhibitor (Venetoclax) with CHOP chemotherapy, highlighting the diagnostic challenges and therapeutic considerations for this aggressive disease.

**Methods:**

We detail the clinicopathological journey of a 72-year-old male patient who presented with an acute abdomen. The diagnosis was established through surgical pathology and comprehensive immunohistochemistry. Following an initial treatment refusal and a subsequent disease recurrence manifesting as a second perforation, the patient received Venetoclax combined with CHOP. The relevant literature was reviewed to inform the diagnostic and therapeutic decisions.

**Results:**

Emergency surgery for perforation revealed MEITL (initially staged as Lugano stage I). The patient initially declined therapy but presented 9 months later with recurrent perforation, confirmed as disease relapse via pathology. Immunohistochemistry showed tumor cells were CD3 (weakly positive), CD8+, CD56+, Bcl-2 (3+), and Ki-67 (~60%). Postoperative PET/CT revealed limited retroperitoneal lymph node involvement. The patient received four cycles of Venetoclax (200 mg/day, days 1–14) plus CHOP, achieving complete metabolic remission on follow-up PET/CT without significant myelosuppression. The 9-month untreated interval despite high Ki-67 is noteworthy.

**Conclusion:**

MEITL is a highly aggressive lymphoma often presenting as a surgical emergency. Diagnosis relies on histopathology and a characteristic immunophenotype (CD3+ CD8+ CD56+). This case suggests that incorporating BCL-2 inhibition into the treatment of Bcl-2–high MEITL may represent a biologically rational strategy. Besides, the prolonged untreated interval observed in this patient highlights the potential biological heterogeneity of MEITL. While the observed complete metabolic remission is encouraging, this finding remains hypothesis-generating and requires validation in larger studies.

## 1 Introduction

Monomorphic epitheliotropic intestinal T-cell lymphoma (MEITL) is an extremely rare and aggressive peripheral T-cell lymphoma derived from intestinal intraepithelial lymphocytes. Recognized as a distinct entity in the 2016 WHO classification, it was formerly categorized as enteropathy-associated T-cell lymphoma (EATL) type II (1). In contrast to EATL type I, MEITL is typically not associated with celiac disease, although rare instances of MEITL occurring in the setting of celiac disease have been documented. This entity exhibits a higher prevalence in Southeast Asian and Hispanic populations, with sporadic reports in China and Western countries (2). Its clinical presentation is non-specific, often leading to emergency surgery for complications like intestinal perforation or obstruction. The prognosis is dismal, with a median overall survival of only 7–10 months, largely due to inherent resistance to conventional anthracycline-based chemotherapy like CHOP (3–5).

Recent molecular studies have elucidated potential mechanisms of this chemoresistance, including frequent mutations in *SETD2*, *STAT5B*, and *JAK3*, which dysregulate JAK/STAT signaling and alter histone methylation (6). Additionally, high expression of anti-apoptotic proteins, such as Bcl-2, has been observed in MEITL, suggesting that it may be a potential therapeutic target (7). The standard of care remains undefined, often involving surgery for emergencies and chemotherapy, with consideration for consolidative hematopoietic stem cell transplantation (HSCT) in eligible patients (8).

This case report details a patient with MEITL presenting with acute perforation, who, after a period of observation and subsequent relapse, achieved complete remission with a novel regimen of Venetoclax (a BCL-2 inhibitor) combined with CHOP. We aim to highlight the diagnostic intricacies of MEITL, discuss the rationale for BCL-2 targeted therapy based on tumor biology, and contribute to the evolving understanding of treatment strategies for this lethal disease.

## 2 Case presentation

### 2.1 Initial presentation and emergency management

A 72-year-old male patient was admitted to the Gastrointestinal Surgery department on December 18, 2024, with a one-day history of diffuse abdominal pain, nausea, and vomiting. Physical examination revealed board-like rigidity, tenderness, rebound tenderness, and diminished bowel sounds. Laboratory tests showed leukocytosis (WBC 10.39 × 10^9^/L) and elevated C-reactive protein (52.69 mg/L). Abdominal CT (Figure 1A) revealed free intraperitoneal gas, confirming gastrointestinal perforation. The patient underwent emergency laparoscopic exploration, which identified a perforated diverticulum approximately 120 cm distal to the Treitz ligament. A partial small bowel resection with primary anastomosis and drainage was performed.

**Figure 1:**
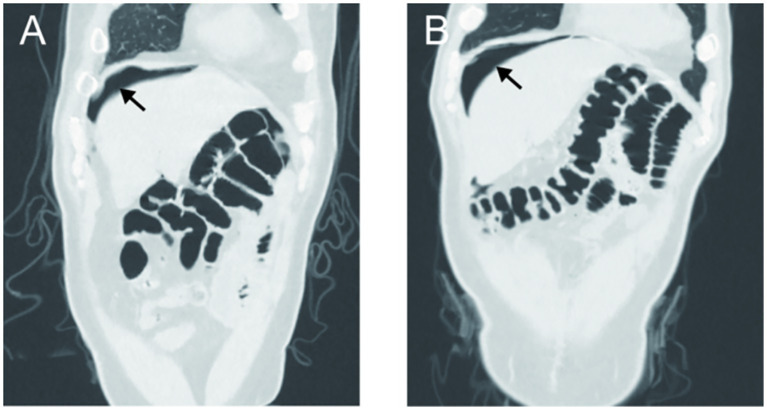
Abdominal CT findings at initial presentation and relapse. **(A)** Abdominal CT performed in December 2024 demonstrating free intraperitoneal air (arrow), consistent with gastrointestinal perforation. **(B)** CT performed in September 2025 showing recurrent free intraperitoneal air (arrow).

### 2.2 Pathological diagnosis of MEITL

Gross Pathology: The resected intestinal segment measured 18 cm. A 3.0 × 0.5 cm perforation was identified, with adjacent mucosal thickening (1.5 × 1.0 cm) and a dark-red serosal surface.

Histopathology (Performed Dec 24, 2024, Figure 2A): Microscopic examination revealed a diffuse infiltration of monomorphic, small-to-medium-sized atypical lymphocytes throughout the bowel wall, with characteristic epitheliotropism (invasion into intestinal crypts).

**Figure 2:**
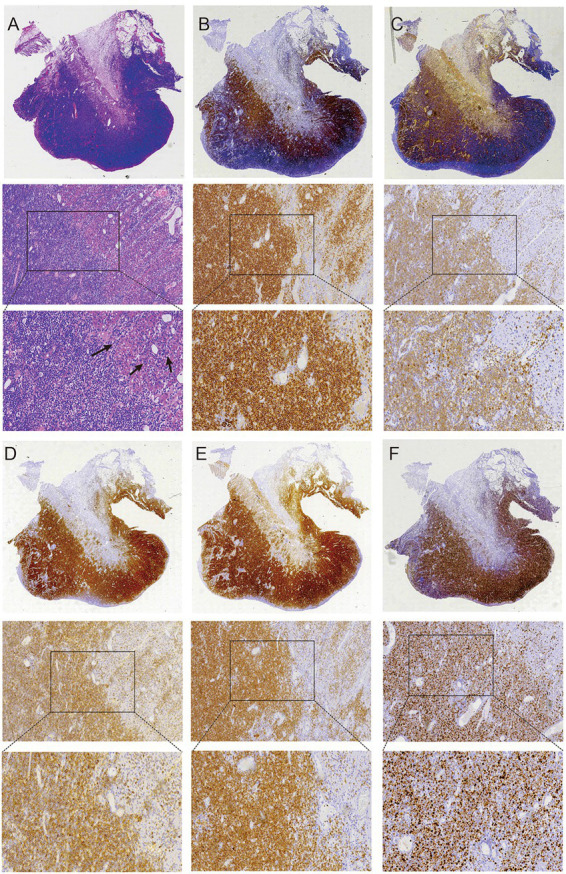
Comprehensive histopathological and immunophenotypic analysis at initial diagnosis. **(A)** Representative H&E sections demonstrating a dense, diffuse infiltration of monomorphic, medium-sized atypical lymphocytes throughout the intestinal wall. The sequence from top to bottom represents the panoramic view, medium-power localization, and high-power magnification. Black arrows in the high-power view highlight the prominent epitheliotropic growth pattern, with tumor cells characteristically infiltrating the glandular epithelium and intestinal crypts. **(B–F)** Immunohistochemical (IHC) profiling across multi-scale views. The neoplastic cells show strong and diffuse positivity for **(B)** CD56, **(C)** CD3, and **(D)** CD8, confirming the T-cell lineage and the specific MEITL phenotype. **(E)** Intense and diffuse overexpression of the anti-apoptotic protein BCL-2 (3+) is observed, providing the biological rationale for subsequent targeted therapy. **(F)** The Ki-67 proliferation index is high at approximately 60%, reflecting the aggressive nature of the malignancy.

Immunohistochemistry (IHC) (Performed Dec 24, 2024; Figures 2B–F): Tumor cells were positive for CD3 (weak), CD43 (3+), CD8 (+), CD56 (3+), TIA-1 (+) and Bcl-2 (3+). They were negative for CD20, CD79a, CD5, CD4, CD30, ALK, EBV (EBER-ISH), and TdT. The proliferation index Ki-67 was approximately 60%. This immunoprofile (CD3+ CD8+ CD56+ CD4- CD5- EBER-) is diagnostic for MEITL. Although next-generation sequencing (NGS) to identify *SETD2* or *JAK3/STAT5B* mutations and T-cell receptor (TCR) gene rearrangement analysis were not performed due to clinical urgency and technical constraints at our center, the diagnosis remains highly robust. The combination of monomorphic cytomorphology, prominent epitheliotropism, and a definitive CD3+ CD8+ CD56+ EBER- immunophenotype strictly fulfills the WHO diagnostic criteria for MEITL.

Histopathology at relapse (Performed Sep 18, 2025, Figure 3A): Microscopic examination revealed a diffuse infiltration of monomorphic, small-to-medium-sized atypical lymphocytes throughout the bowel wall, with characteristic epitheliotropism (invasion into intestinal crypts).

**Figure 3:**
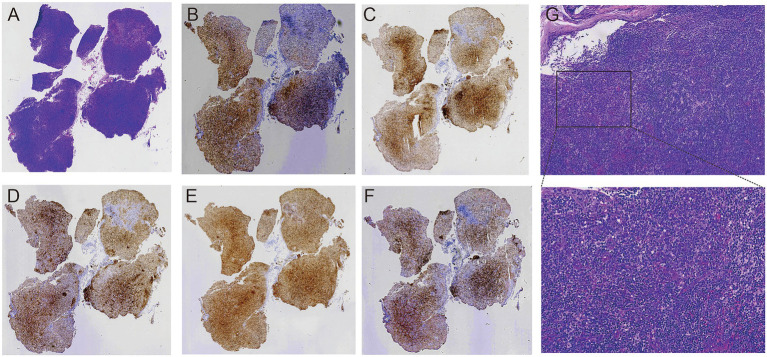
Histopathological and immunophenotypic evaluation of the relapsed tumor (September 2025). **(A)** Low-power panoramic view of the H&E stained section from the second surgical specimen, showing dense transmural lymphoid infiltration. **(G)** Progressively magnified views of the area indicated by the black box in the H&E section. The high-power magnification confirms the recurrence of monomorphic, medium-sized atypical lymphocytes with extensive and diffuse epitheliotropism. Given the widespread nature of the neoplastic infiltration throughout the intestinal epithelium, specific arrow indicators were omitted to better maintain the visual clarity of the overall cellular morphology and the characteristic permeation pattern, which remains consistent with the primary tumor. **(B–F)** Immunohistochemistry profiling of the relapsed tissue. The neoplastic cells maintain an identical immunophenotype to the initial diagnosis, showing positive for **(B)** CD56, **(C)** CD3, **(D)** CD8, **(E)** Bcl-2 (3+), and **(F)** Ki-67 (~60%).

IHC at relapse (Performed Sep 18, 2025; Figures 3B–F): Tumor cells were positive for CD3 (weak), CD43 (3+), CD8 (+), CD56 (3+), TIA-1 (+) and Bcl-2 (3+). The proliferation index Ki-67 was approximately 60%.

Diagnosis and Staging: A diagnosis of MEITL was rendered. Postoperative bone marrow biopsy was negative, with no involvement. The disease was initially staged as Lugano stage I (localized disease). At the initial diagnostic stage, comprehensive clinical and hematological staging was performed. Contrast-enhanced CT scans of the chest, abdomen, and pelvis, combined with a negative bone marrow aspiration and biopsy, confirmed that the lymphoma was primary and localized to the small bowel, with no evidence of distant metastasis or extra-intestinal involvement. The patient was informed of the diagnosis and poor prognosis, but declined any further adjuvant therapy at that time.

### 2.3 Disease recurrence and re-assessment

On September 9, 2025, the patient presented again with acute abdominal pain. CT (Figure 1B) showed recurrent free air, consistent with a new perforation. He underwent a second surgery (abdominal irrigation, drainage, and enterostomy). Pathological examination (Figure 3) of the new surgical specimen confirmed recurrent lymphoma with identical morphological and immunophenotypic features, indicating a relapse rather than a new primary event. Besides, no evidence of an alternative surgical complication (e.g., anastomotic leakage/ischemia) was identified on pathology, supporting true relapse.

At relapse, restaging PET/CT conducted in November 2025 (Figure 4A) demonstrated newly developed fluorodeoxyglucose (FDG)-avid retroperitoneal lymphadenopathy (SUVmax 15.2), indicating nodal involvement and radiologic progression. The diagnosis was reaffirmed by an external pathology review at Ruijin Hospital (Shanghai). The expert consultation confirmed the characteristic monomorphic lymphoid infiltration and the specific immunophenotypic profile. While the external report used descriptors such as “weak” or “suboptimal” for the intensity of certain markers (CD3, CD8, and CD56) compared to the initial evaluation, these differences reflect the inherent inter-observer subjectivity in assessing staining intensity. Crucially, both institutions concurred on the positive/negative status of the key markers and their characteristic distribution pattern (CD3+ CD8+ CD56+ EBER-), which collectively fulfill the diagnostic criteria for MEITL.

**Figure 4:**
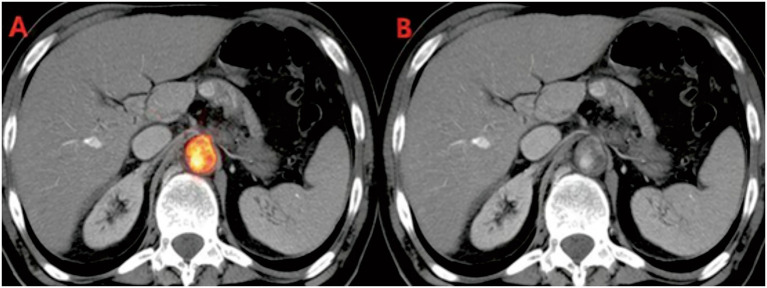
PET/CT imaging for staging and response assessment. **(A)** Restaging PET/CT performed in November 2025 demonstrating FDG-avid retroperitoneal lymphadenopathy (SUVmax 15.2, arrow). **(B)** Follow-up PET/CT performed in January 2026 showing complete metabolic resolution.

### 2.4 Exploratory treatment with Venetoclax + CHOP and follow-up

To provide a structured overview of the clinical journey—encompassing the initial surgical emergency, the prolonged untreated interval, disease relapse, and the subsequent cycles of targeted therapy—a detailed Timeline of Clinical Events is presented in Table 1. Given the disease progression, the patient’s Bcl-2 (3+) expression, and recent literature suggesting potential efficacy of BCL-2 inhibition in T-cell malignancies, an exploratory regimen was proposed. After multidisciplinary discussion and obtaining informed consent, treatment with Venetoclax plus CHOP was initiated on November 13, 2025.

**Table 1:** Timeline of diagnosis, treatment, and response.

Date	Clinical event	Diagnostic findings/therapy	Outcome/response
Dec 18, 2024	Initial presentation with acute abdomen	CT: GI perforation. Emergency surgery	Surgery successful
Dec 24, 2024	Pathological diagnosis	IHC confirms MEITL (Lugano I)	Patient declines therapy
Sep 9, 2025	Disease recurrence	CT: recurrent perforation. surgery	Pathology confirms relapse
Nov 12, 2025	Restaging	PET/CT: Retroperitoneal LN (SUVmax 15.2)	Confirmed progression
Nov 13, 2025	Cycle 1 therapy	Venetoclax (200 mg d1-14) + CHOP	Well tolerated
Dec 4, 2025	Cycle 2	Venetoclax + CHOP	Well tolerated
Dec 25, 2025	Cycle 3	Venetoclax + CHOP	Well tolerated
Jan 16, 2026	Cycle 4	Venetoclax + CHOP	Well tolerated
Jan 28, 2026	End-of-treatment evaluation	PET/CT: complete metabolic remission	CMR achieved
Apr 2026	Routine follow-up	/	Sustained CMR confirmed

Treatment regimen (Table 1): Venetoclax was administered at a dose of 200 mg orally daily on days 1–14 of each 21-day cycle, based on a reduced-dose schedule accounting for the patient’s age and to mitigate the risk of tumor lysis syndrome (TLS) and severe myelosuppression reported with higher doses in T-cell lymphomas (9). During the first week of treatment, the patient was closely monitored for TLS through daily assessments of serum electrolytes, uric acid, and renal function. Notably, no clinical or laboratory evidence of TLS was observed. This was combined with standard-dose CHOP (cyclophosphamide, doxorubicin, vincristine, prednisone).

#### 2.4.1 Outcome

The patient tolerated four cycles well without significant myelosuppression or other major complications. A follow-up PET/CT performed on January 28, 2026 (Figure 4B), demonstrated complete metabolic remission (CMR), with resolution of the previously noted lymphadenopathy. As of April 2026, the patient has maintained continuous clinical and metabolic remission for 6 months following the completion of the exploratory regimen. He continues to undergo monthly clinical surveillance and scheduled radiological reassessments. While the current CMR is encouraging, the long-term prognosis remains guarded due to the high risk of late recurrence inherent to MEITL, necessitating lifelong vigilance.

## 3 Discussion

This case illustrates the formidable diagnostic and therapeutic challenges posed by MEITL. The patient’s initial presentation with life-threatening perforation is classic for this entity, which often eludes pre-operative diagnosis due to non-specific symptoms (10). The definitive diagnosis was based on the histopathological triad of monomorphic lymphoid infiltration, epitheliotropism, and the characteristic immunophenotype (CD3+ CD8+ CD56+ CD4- CD5- EBER-), effectively excluding mimics such as EATL type I, extranodal NK/T-cell lymphoma, and indolent T-cell lymphoproliferative disorders of the GI tract (1, 11).

### 3.1 Differential diagnosis

The differential diagnosis of primary intestinal T-cell lymphomas is clinically and pathologically challenging due to overlapping features (Table 2). In our case, the monomorphic cytomorphology and the characteristic immunophenotype—specifically the strong expression of CD8 and CD56, along with the absence of CD5 and CD30—strongly supported the diagnosis of MEITL over other subtypes.

**Table 2:** Differential diagnosis of primary intestinal T-cell lymphomas.

Feature	MEITL (present case)	EATL Type I	EBV+ NK/T-cell lymphoma	Indolent T-cell LPD of GI tract
Epidemiology	Asia/Hispanic, M > F	Celiac disease, Caucasian	Asia, Latin America	Western, M = F
Cell size	Small to medium, monomorphic	Large, pleomorphic	Variable, often angioinvasive	Small, mature
CD8	±	−(Usually)	−/+	−/+
CD56	+(Strong)	−/+(Weak)	±	−
CD5	−	−	−	±
EBER	−	−	±	−
CD30	−	±	Variable	−
TCR	*γ*/*δ* (often)	*α*/*β*	*γ*/*δ* or *α*/*β*	*α*/*β*

TCR analysis was not performed for the present case.

Specifically, enteropathy-associated T-cell lymphoma (EATL) type I was excluded due to the lack of celiac disease history and the absence of large, pleomorphic cells. Extranodal NK/T-cell lymphoma was ruled out by the negative EBER-ISH result, which is a mandatory exclusion criterion for MEITL. Furthermore, the aggressive clinical course, characterized by recurrent intestinal perforations and a high Ki-67 index, easily distinguished this case from indolent T-cell lymphoproliferative disorders of the gastrointestinal tract, which typically exhibit a mature T-cell phenotype (CD5+) and a far more favorable prognosis.

It is well-recognized that the majority of MEITL cases express the *γ*/*δ* T-cell receptor (TCR), while a minority are of *α*/βT-cell origin. Although TCR gene rearrangement analysis or γ/δ IHC was not performed in our case due to institutional technical limitations at the time of diagnosis, the diagnosis of MEITL remains highly robust. According to the WHO classification of haematolymphoid tumors, the combination of classic monomorphic cytomorphology, a prominent epitheliotropic growth pattern, and the characteristic CD3+/CD8+/CD56+ immunophenotype is sufficient to establish a definitive diagnosis of MEITL.

### 3.2 Diagnostic nuances and the untreated interval

The 9-month interval from initial diagnosis to disease recurrence observed in this case is highly unconventional. MEITL is notoriously aggressive, carrying a dismal prognosis with median overall survival often limited to 7–10 months (3, 4). A nearly year-long period of stability following surgical intervention alone warrants consideration. This clinical outlier likely resulted from the identification of the disease at an exceptionally early stage (Lugano Stage I), where the emergency surgery achieved a complete macroscopic (R0) resection of the localized primary lesion. By effectively debulking the predominant tumor load, the surgical intervention may have temporarily halted the immediate progression.

Furthermore, this prolonged dormant period may reflect the biological heterogeneity inherent to MEITL. Although the Ki-67 index was high (~60%), the tumor’s growth kinetics in its localized phase might be less explosive in certain molecular subsets compared to its behavior during systemic dissemination. Nevertheless, the eventual recurrence as a second perforation and retroperitoneal nodal involvement confirms that this stability was transient. This clinical trajectory serves as a critical reminder that the aggressive nature of MEITL remains its defining feature; even after successful surgical management of early-stage disease, immediate systemic therapy is essential to prevent inevitable and catastrophic relapse.

### 3.3 Rationale for BCL-2 inhibition in MEITL

The decision to employ Venetoclax was biologically guided by the patient’s specific protein expression profile. Molecularly, MEITL is characterized by a convergent pathogenic axis involving deregulated histone methylation (SETD2 loss-of-function) and constitutive JAK–STAT pathway activation, predominantly through *JAK3* and *STAT5B* mutations importantly, constitutive STAT5 activation has been shown to directly upregulate the expression of anti-apoptotic BCL-2 family proteins, providing a mechanistic link between the disease’s driver mutations and BCL-2 protein abundance.

In the present case, although NGS was not performed due to clinical constraints and the patient’s initial hesitation, the diffuse and intense BCL-2 expression (3+) observed via immunohistochemistry served as a reliable surrogate for the activated JAK–STAT/BCL-2 signaling axis. Notably, the re-biopsy from the second perforation site not only confirmed the histological relapse but also verified the sustained, strong expression of Bcl-2 (3+). This persistent biomarker profile across the disease progression provided the ultimate rationale for our subsequent targeted therapy, highlighting the importance of re-evaluating tumor biology in the setting of recurrence. While MEITL is a T-cell lymphoma, aberrant overexpression of Bcl-2 is a recognized mechanism of chemoresistance. Preclinical and early clinical data suggest that subsets of peripheral T-cell lymphomas (PTCL), including those with specific genetic alterations, may be dependent on Bcl-2 for survival (9, 11). By targeting the “molecular vulnerability” with Venetoclax, we were able to sensitize the refractory tumor cells to chemotherapy and bypass conventional resistance, ultimately achieving complete metabolic remission.

Venetoclax has shown activity in relapsed/refractory T-cell lymphomas, but its use is often limited by hematological toxicity, particularly when combined with chemotherapy (12). We employed a cautious dose (200 mg/day for 14 days per cycle) instead of the higher doses used in B-cell malignancies, aiming to balance efficacy and safety in an elderly patient. The successful outcome without severe myelosuppression suggests this modified schedule may be a viable approach, contrasting with a reported fatal outcome using a higher dose (400 mg/day) in a similar context (13).

While the combination of Venetoclax and CHOP is not currently the standard of care for MEITL, our decision was supported by emerging evidence in other T-cell malignancies. For instance, Ballotta et al. (9) recently demonstrated the therapeutic potential of Venetoclax in BCL2-positive relapsed/refractory peripheral T-cell lymphomas. In the current case, the sustained and intense BCL-2 (3+) expression served as a crucial predictive biomarker, justifying this exploratory therapeutic approach. This regimen should be regarded as experimental in the context of MEITL, specifically aimed at overcoming the intrinsic chemoresistance of the disease.

### 3.4 Therapeutic context and future directions

Our case adds to the growing anecdotal evidence that adding Venetoclax may overcome some of this resistance in Bcl-2-high tumors. However, this remains an exploratory approach. For eligible patients, consolidative autologous or allogeneic HSCT after remission induction is considered a strategy to improve long-term outcomes and should be pursued when possible (8). Our patient’s age and comorbidities precluded this option.

Future management of MEITL will likely rely on molecular profiling to guide targeted therapy. Beyond BCL-2 inhibition, agents targeting the JAK/STAT pathway (e.g., JAK inhibitors) or epigenetic modifiers show promise given the mutational landscape of MEITL (6, 14). This case underscores the need for prospective studies to validate biomarker-driven therapies in this rare and lethal disease.

## 4 Conclusion

MEITL is a diagnostic mimic of common surgical abdomens, requiring a high index of suspicion and reliance on definitive pathology. This case demonstrates that a biologically rational combination of Venetoclax with CHOP can achieve complete metabolic remission in an individual patient with Bcl-2–high MEITL. While encouraging, this observation remains exploratory and hypothesis-generating, and the benefit has yet to be established in larger cohorts. The observed prolonged interval before progression highlights the disease’s heterogeneity. Multidisciplinary collaboration, accurate pathologic characterization, and exploration of molecularly informed therapies are crucial to improving outcomes for patients with this aggressive lymphoma.

This report represents a single case with limited follow-up duration. While the observed complete metabolic remission is encouraging, the use of Venetoclax in MEITL remains exploratory. Larger studies and molecularly stratified analyses are required to determine whether Bcl-2 expression may serve as a predictive biomarker in this context.

## Data Availability

The original contributions presented in the study are included in the article/supplementary material, further inquiries can be directed to the corresponding author.
